# Antioxidant Activity of an Aqueous Extract of Cuttlefish Ink during Fish Muscle Heating

**DOI:** 10.3390/antiox12111996

**Published:** 2023-11-13

**Authors:** Marcos Trigo, David Paz, Antía Bote, Santiago P. Aubourg

**Affiliations:** Department of Food Technology, Marine Research Institute (CSIC), c/E. Cabello, 6, 36208 Vigo, Spain; mtrigo@iim.csic.es (M.T.); david.paz.perez@alumnos.uvigo.es (D.P.); antia.bote.chamorro@alumnos.uvigo.es (A.B.)

**Keywords:** cuttlefish ink, seabream muscle, heating, lipid oxidation, lipid hydrolysis, fatty acid profile, polyene content, antioxidant behaviour

## Abstract

The antioxidant effect of cuttlefish (*Sepia officinalis*) ink (CFI) was analysed in the present study. A model system consisting of minced seabream (*Sparus aurata*) muscle and different concentrations of an aqueous extract of CFI was subjected to a heat (50 °C) treatment for 12 days. The effects of the CFI content and the heating time on lipid oxidation (conjugated diene (CD), conjugated triene (CT), and peroxide values and fluorescent compound formation), hydrolysis (free fatty acid content) development, and changes in the fatty acid (FA) profile (polyene index (PI), unsaturated FA content, ω3/ω6 ratio) were determined. The addition of the aqueous extract of CFI led to a lower (*p* < 0.05) development of lipid oxidation (CD, CT, and fluorescent compound determination) and to a higher (*p* < 0.05) retention of unsaturated FAs (PI determination). More important effects were found with increased CFI concentrations and at advanced heating times. However, a definite effect on lipid hydrolysis development (FFA value) could not be inferred. A new approach for the beneficial use of cuttlefish ink is presented. According to the direct relationship between rancidity stability and nutritional and sensory values, the present study provides a new strategy for the quality enhancement of thermally treated seafood.

## 1. Introduction

Seafood is reported to provide a high amount of constituents relevant for the human diet [1]. Among such components, the lipid fraction is currently the subject of considerable attention due to its remarkable ω3 polyunsaturated FA (PUFA) content and its valuable role in preventing the development of several human diseases [2,3]. However, due to the marine nature of their constitution, seafood products are perishable and can undergo a wide range of detrimental effects during processing, such as microbial development, lipid oxidation, autolysis, and browning development [4,5]. The modification of marine constituents can be especially important when thermal treatments are involved as such treatments can lead to lipid and vitamin oxidation, nutrient degradation, and loss of vitamins, minerals, and proteins [6,7].

The employment of compounds with antioxidant properties has revealed them to be valuable tools for inhibiting or partially avoiding the development of lipid oxidation in seafood. Due to human health concerns, there is currently great interest in replacing synthetic antioxidants with preservative compounds obtained from natural sources [8,9]. Therefore, a wide range of natural antioxidants has been shown to produce relevant preservative effects on different kinds of marine substrates (i.e., oils and muscle) during thermal processing [10,11,12,13].

Cephalopods are considered a remarkable economic resource in a wide range of countries. As for other marine species, a great number of by-products (head, skin, viscera, etc.) are generated during their technological processing [14,15]. In addition to their main constituents (proteins and lipids), the by-products of seafood in general have been reported to include valuable and healthy minor constituents such as enzymes, vitamins, minerals, and amino acids [16,17]. The utilisation of by-products would not only benefit the industry, but it would also prevent or partially inhibit environmental problems and pollution.

Among cephalopod by-products, ink sacs are commonly removed manually and thrown away without appropriate management. However, recent studies on this kind of by-product have revealed that they possess several properties beneficial to human health, such as a therapeutic effect against invasive pulmonary aspergillosis [18], suitability for use in photothermic therapy [19,20], and suitability for biomedical applications related to antioxidant, chemical, and theranostic therapies [19]. Furthermore, cuttlefish ink has been incorporated into both eye cosmetic products [21] and a polyamidoxime-adsorbent system for the efficient capture of uranium from natural seawater [22]. Regarding the preservation of seafood, previous studies have shown that cephalopod ink extracts possess relevant antimicrobial properties that act against a wide range of pathogenic strains [23,24,25] as well as valuable antioxidant properties when applied to different kinds of seafood substrates [26,27,28] or incorporated into novel packing systems [29].

The present study is focussed on the use of cuttlefish (*Sepia officinalis*) ink. The basic objective was to analyse its antioxidant behaviour and properties in a heated fish muscle system. Complementary quality indices regarding the lipid damage (lipid oxidation, hydrolysis development, and changes in the FA profile) were measured during a heating process (50 °C for 12 days) carried out with minced seabream (*Sparus aurata*). The effects of the CFI concentration and the heating time were analysed.

## 2. Materials and Methods

### 2.1. Raw Fish, Starting Ink, and Preparation of the Ink Extract

Seabream (*S. aurata*) (6 specimens, ca. 500 g each) were obtained in the fresh state from a local market and transported on ice to the laboratory. The fish specimens were then randomly divided into three (*n* = 3) groups (2 specimens per group). Within each group, the specimens were beheaded, eviscerated, and filleted; finally, the white muscle of the fish fillets was removed, minced, and homogenised.

Commercial cuttlefish (*S. officinalis*) ink was provided by Sepink (Vilagarcía de Arousa, Spain). The ink exhibited the following proximate composition (g·100 g^−1^): 78.4 (moisture), 6.4 (protein), 0.1 (lipids), 9.0 (ash), and 5.6 (total fibre).

To prepare the cuttlefish ink (CFI) extract, a mixture of CFI (6 g) and distilled water (100 mL) was stirred (30 s), sonicated (30 s), and centrifuged (3500× *g* for 30 min at 4 °C). The resulting supernatant was then collected. This extraction process was repeated three more times. Finally, all four supernatants were pooled together and made up to 250 mL with distilled water.

In the present study, an aqueous extract of CFI was employed. This choice was based on the results of previous studies which have shown that aqueous ink extracts obtained from different cephalopod substrates, such as splendid squid (*Loligo formosana*) [27,28] and cuttlefish [29], exhibit remarkable antioxidant activity during seafood processing. Furthermore, aqueous extracts obtained from other marine substrates have also exhibited valuable antioxidant properties during seafood processing. In studies of brown [12,30], green [12], and red [13] macroalgae, proteins, peptides, and especially polysaccharides were reported to be responsible for antioxidant behaviour. Additionally, the use of water instead of organic solvents is currently recommended because it is considered an eco-friendly procedure.

### 2.2. Ink Extract/Seabream Muscle System

For the preparation of the minced fish system, fish muscle portions corresponding to the above-mentioned homogenised groups were employed. In this system, 4 g portions of seabream muscle were mixed with 0, 2, 5, or 10 mL of the above-mentioned CFI extract. Next, 10, 8, 5, or 0 mL of distilled water was added to each respective mixture, resulting in Control (CTR), CFI-1 (low CFI concentration), CFI-2 (medium CFI concentration), and CFI-3 (high CFI concentration) batches.

Mixtures corresponding to all batches were stirred (30 s), sonicated (30 s), and incubated (50 °C for 12 days). Sampling was carried out at 0, 2, 5, 8, and 12 days of the heating time. At each sampling time, mixtures were cooled to room temperature (18–20 °C). Then, mixtures were centrifuged, the supernatant discarded, and the solid phase (i.e., the seabream muscle) subjected to the extraction of the lipid fraction. The study was carried out by means of three independent sets that were analysed independently (*n* = 3). Additionally, each chemical analysis was carried out in triplicate for each single sample.

### 2.3. Assessment of Lipid Damage

The lipid fraction was obtained by extraction of the seabream muscle by employing the Bligh and Dyer [31] method; this procedure uses a single-phase solubilisation of the lipids by employing a chloroform–methanol (1:1) mixture. Lipid content is expressed as g·kg^−1^ seabream muscle.

The formation of conjugated diene (CD) compounds was determined spectrophotometrically (Beckman Coulter DU 640, Spectrophotometer, Beckman Coulter Inc., Brea, CA, USA) at 233 nm [32] on the lipid fraction. The results were calculated according to the formula: CD = *A·V·w*^−1^, where *A* is the resulting spectrophotometric reading and *V* and *w* indicate the volume (mL) and the weight (mg) of the lipid extract measured, respectively.

The formation of conjugated triene (CT) compounds was measured at 268 nm [32] on the lipid fraction. The results were calculated according to the formula: CT = *A·V·w*^−1^, where *A* is the resulting spectrophotometric reading and *V* and *w* indicate the volume (mL) and the weight (mg), respectively, of the lipid extract measured.

The assessment of the peroxide value (PV) was carried out spectrophotometrically (520 nm) on the lipid fraction. For it, peroxide reduction with ferric thiocyanate was carried out [33]. The results are expressed as meq active oxygen·kg^−1^ lipids.

The assessment of the free fatty acid (FFA) content was carried out on the lipid extract of the seabream muscle following the Lowry and Tinsley [34] method. This method is based on the complex formation with cupric acetate-pyridine followed by spectrophotometric (715 nm) determination. The results are expressed as mg FFA·kg^−1^ muscle.

### 2.4. Determination of Interaction Compound Formation

The determination of the interaction compound formation produced by the reaction of oxidised lipids and protein-type molecules was carried out by fluorescence spectroscopy (Fluorimeter LS 45; Perkin Elmer España; Tres Cantos, Madrid, Spain). Fluorescent compound formation was measured at 393/463 and 327/415 nm [35]. The relative fluorescence (RF) was calculated according to the formula: RF = *F/F_st_*, where *F* is the fluorescence measured at each excitation/emission wavelength pair and *F_st_* is the fluorescence intensity of a quinine sulphate solution (1 µg·mL^−1^ in 0.05 M H_2_SO_4_) at the corresponding wavelength pair. The formation of fluorescent compounds was calculated as the fluorescence ratio (FR) between the two RF values: FR = RF_393/463 nm_/RF_327/415 nm_.

To obtain a more complete detection of the interaction compound formation, two different substrates obtained from the ink extract/minced seabream system were considered. Thus, the fluorescence ratio was analysed in the organic (FR_org_) and aqueous (FR_aq_) phases obtained from the lipid extraction [31] of the fish muscle.

### 2.5. FA Profile Analysis

Lipid extracts were converted into fatty acid methyl esters (FAMEs) by employing an acid-catalysed esterification and transesterification method (i.e., acetyl chloride in methanol) [30]. Then, the FAMEs obtained were analysed by gas–liquid chromatography (Perkin Elmer 8700 chromatograph, Madrid, Spain). The qualitative analysis was undergone in agreement with the FAME retention times and comparison to standard mixtures (Qualmix Fish, Larodan, Malmo, Sweden; FAME Mix, Supelco, Inc., Bellefonte, PA, USA). For quantitative purposes, peak areas were automatically integrated, and C19:0 FA was employed as an internal quantitative standard. The presence of each FA is expressed as g·100 g^−1^ total FAs.

The results regarding FA groups (saturated FAs (STFAs), monounsaturated FAs (MUFAs), polyunsaturated FAs (PUFAs), ω3 and ω6 FAs) and FA ratios (C20:5ω3 + C22:6ω3/C16:0, polyene index (PI), total MUFAs + total PUFAs/total STFAs, total ω3 FAs/total ω6 FAs) were calculated considering the values obtained for individual FAs.

### 2.6. Statistical Analysis

Values obtained for the different response or dependent variables (CDs, CTs, PV, FR_org_, FR_aq_, FFAs, and FA ratios) were subjected to the multifactorial ANOVA method (randomised-block design) to analyse differences as a result of two process or independent variables (the CFI concentration and the heating time) [36]; the ANOVA analysis showed that both independent variables provided significant differences (*p* < 0.05). As expressed above, three independent groups (i.e., three replicates, *n* = 3) were taken into account in the present research. To carry out the comparison of means, the Fisher’s least-squares difference (LSD) method was employed. Statistical analysis was determined with the PASW Statistics 18 software for Windows (SPSS Inc., Chicago, IL, USA). The 95% confidence intervals of each lipid parameter were calculated; for this calculation, the standard deviation of each sample and the number of replicates were considered.

Correlation analyses between lipid damage values and the heating times were performed according to the Pearson method. Correlation values corresponding to linear fittings are expressed; otherwise, the kind of fitting is expressed.

## 3. Results

### 3.1. Determination of Lipid Oxidation

The evolution of the CD content during the heating time is shown in Table 1.

In most cases, a progressive formation (*p* < 0.05) with heating time was detected for the CD content (*r* = 0.83–0.90, logarithmic fitting). A comparison among batches revealed an inhibitory effect (*p* < 0.05) on the formation of this kind of oxidised lipids as a result of the presence of the CFI in the heating medium. This effect was shown to increase with the CFI concentration. Thus, fish muscle corresponding to the CFI-3 batch showed lower (*p* < 0.05) CD levels than its counterpart CTR batch for the 2–8-day period.

The formation of CTs also showed a progressive increase during the whole heating time (*r* = 0.87–0.93) (Table 1). An increase (*p* < 0.05) in CTR, CFI-1, CFI-2, and CFI-3 batches was detected after 5, 8, 12, and 12 days of the heating time, respectively. As for the CD formation, the presence of the CFI in the reacting medium led to lower average CT values. Notably, differences between the CTR batch and those including the two highest CFI concentrations (i.e., CFI-2 and CFI-3 batches) were found significant for the 5–8-day period.

Peroxide formation in heated fish lipids can be observed in Table 1. As a general behaviour, a progressive formation was detected in all batches up to a certain heating time that was followed by a progressive decrease until the end of the experiment. In the case of the CTR batch, the highest (*p* < 0.05) PV was observed after 5 days of heating time. In the case of the CFI-treated batches, the highest peroxide contents (*p* < 0.05) were observed after an 8-day heating time. A comparison among batches showed the highest PV in control canned fish for the 2–5-day period. Later on (8–12-day period), the highest mean values were observed in fish samples corresponding to the CFI-1 batch, and the lowest ones were detected in fish corresponding to the CFI-3 batch.

### 3.2. Determination of Fluorescent Compound Formation

**Figure 1:**
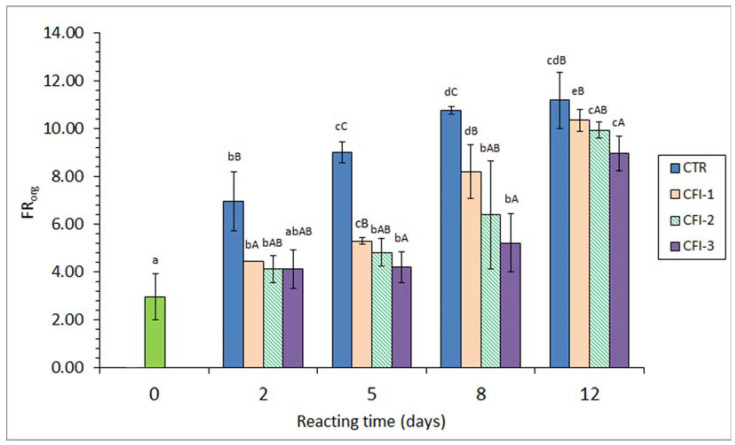
Fluorescence ratio (FR) in the lipid extract (FR_org_) of seabream muscle heated in the presence of an aqueous extract of cuttlefish ink (CFI). Mean values of three replicates (*n* = 3); standard deviations are shown by bars. Different lowercase letters (a–e) denote differences (*p* < 0.05) as a result of the heating time; different capital letters (A–C) denote differences (*p* < 0.05) as a result of the CFI concentration. Abbreviations as expressed in Table 1.

A marked fluorescence formation (*p* < 0.05) in the lipid extract (i.e., FR_org_) was detected in all batches as a result of increasing the heating time (*r* = 0.86–0.94) (Figure 1).

Additionally, the highest mean levels were observed in all cases at the end of the experiment. A comparison among batches revealed lower mean values in the CFI-treated muscle than in their counterparts corresponding to the CTR condition. Thus, all tested CFI conditions led to lower values (*p* < 0.05) for the 5–8-day period when compared to fish muscle subjected to the CTR condition. Additionally, fish corresponding to the CFI-1 and CFI-3 batches showed lower (*p* < 0.05) values on days 2 and 15, respectively, than their counterpart CTR samples.

**Figure 2:**
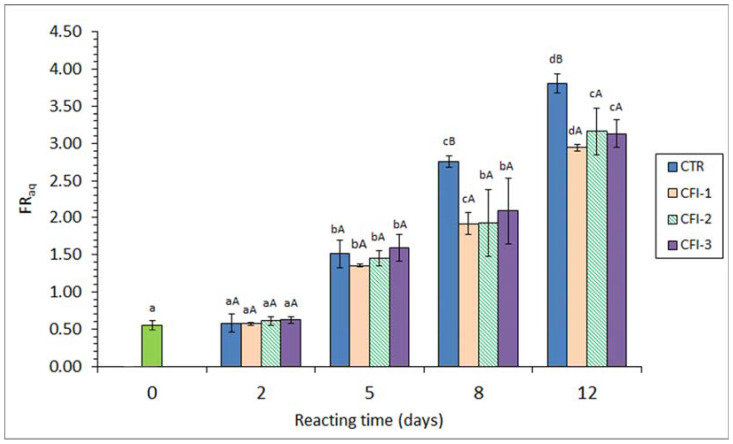
Fluorescence ratio (FR) in the aqueous phase (FR_aq_) resulting from the lipid fraction of seabream muscle heated in the presence of an aqueous extract of cuttlefish ink (CFI). Mean values of three replicates (*n* = 3); standard deviations are shown by bars. Different lowercase letters (a–d) denote differences (*p* < 0.05) as a result of the heating time; different capital letters (A,B) denote differences (*p* < 0.05) as a result of the CFI concentration. Abbreviations as expressed in Table 1.

Data concerning the FR in the aqueous phase resulting from the lipid extraction (i.e., FR_aq_) are shown in Figure 2. No effect (*p* > 0.05) after 2 days of heating treatment could be observed in any of the batches considered. However, a progressive increase (*p* < 0.05) with heating time was detected in all kinds of batches for the 5–12-day period (*r* = 0.93–0.95). A comparison among batches showed higher (*p* < 0.05) values in the CTR fish than in any of the CFI-treated samples during the 8–12-day heating time. The lowest mean levels were observed in fish corresponding to the CFI-1 batch for all the heating time.

### 3.3. Determination of Lipid Hydrolysis

**Figure 3:**
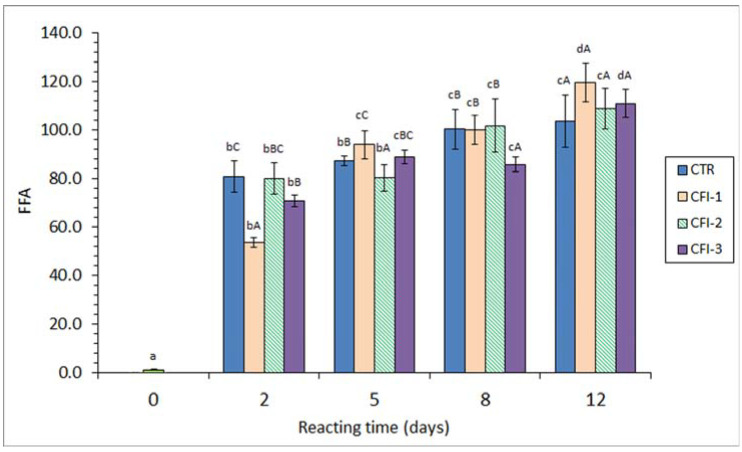
Free fatty acid (FFA) content (mg·kg^−1^ muscle) in the lipid extract of seabream muscle heated in the presence of an aqueous extract of cuttlefish ink (CFI). Average values of three replicates (*n* = 3); standard deviations are shown by bars. Different lowercase letters (a–d) denote differences (*p* < 0.05) as a result of the heating time; different capital letters (A–C) denote differences (*p* < 0.05) as a result of the CFI concentration. Abbreviations as expressed in Table 1.

A strong FFA formation (*p* < 0.05) was detected in fish muscle corresponding to all batches. A remarkable correlation value with the heating time (*r* = 0.88–0.94, logarithmic fitting) was observed (Figure 3). In all cases, this increase was especially important (*p* < 0.05) after a 2-day heating time. A comparison among batches showed some differences (*p* < 0.05); however, a definite trend in the presence and concentration of the CFI in the heating medium could not be concluded. Compared to the CTR fish, an inhibitory effect (*p* < 0.05) on the FFA content was observed on day 2 (CFI-1 and CFI-3 batches), day 5 (CFI-2 batch) and day 8 (CFI-3 batch). Contrarily, fish muscle corresponding to the CTR batch showed the lowest average values at the end of the experiment.

### 3.4. Analysis of the FA Profile

The FA profile of the starting fish muscle showed the following composition (g·100 g^−1^ total FAs): 1.96 ± 0.01 (C14:0), 0.27 ± 0.02 (C15:0), 15.39 ± 0.07 (C16:0), 3.53 ± 0.02 (C16:1ω7), 0.37 ± 0.01 (C17:0), 4.32 ± 0.05 (C18:0), 31.73 ± 0.05 (C18:1ω9), 3.60 ± 0.01 (C18:1ω7), 24.38 ± 0.08 (C18:2ω6), 1.28 ± 0.01 (C20:1ω9), 0.87 ± 0.03 (C20:2ω6), 0.83 ± 0.06 (C20:4ω6), 0.32 ± 0.02 (C22:1ω9), 2.38 ± 0.03 (C20:5ω3), 0.26 ± 0.01 (C22:4ω6), 0.39 ± 0.02 (C24:1ω9), 1.86 ± 0.02 (C22:5ω3), and 6.27 ± 0.09 (C22:6ω3). When FA groups were taken into account, the following composition was observed (g·100 g^−1^ total FAs): 22.31 ± 0.08 (STFAs), 40.85 ± 0.11 (MUFAs), 36.84 ± 0.06 (PUFAs), 10.51 ± 0.09 (total ω3 FAs), 26.34 ± 0.05 (total ω6 FAs), 0.56 ± 0.01 (PI), 3.48 ± 0.09 (MUFA + PUFA/STFA ratio), and 0.40 ± 0.00 (ω3/ω6 ratio).

Recent research has given great attention to certain FA groups according to their valuable properties. Among them, the total content of PUFAs included in the human diet has been signalled as being directly related to nutritional content, digestibility, and preserving properties (i.e., antioxidant and anti-inflammatory) [37,38]. Meantime, great interest is being accorded to the total ω3/total ω6 ratio, based on its important role in the development of cardiovascular, neurological, and inflammatory disorders [39,40]. Taking into account these considerations and on the basis of previous studies carried out on thermally treated seafood [41,42,43,44], the following discussion in this manuscript will be focussed on the PUFA content and on the ω3/ω6 ratio. For it, the PI (EPA + DHA/C16:0 ratio) and the MUFA + PUFA/STFA and ω3/ω6 FA ratios will be analysed.

The heating time led to very scarce changes in the PI of the fish muscle corresponding to all batches (Table 2). In all cases, values were included in a narrow range (i.e., 0.53–0.57). A slight decreasing trend in the PI with an increase in the reaction time could be observed for the CTR and CFI-1 batches; this effect was found significant (*p* < 0.05) for the CTR batch. A comparison among batches showed a protective effect (*p* < 0.05) on PUFAs on days 5 (CFI-2 batch) and 8 (CFI-3 batch) as a result of the CFI presence.

The analysis of the two other above-mentioned FA ratios (i.e., MUFA + PUFA/STFA and ω3/ω6) revealed no effect (*p* > 0.05) of the heating time and the CFI presence and concentration in the heating medium. Thus, values for both ratios were included in very narrow ranges (3.42–3.49 and 0.39–0.41, respectively). It is worth pointing out that the CTR batch did not show differences (*p* > 0.05) for both ratios during the heating time. Similarly, no effect (*p* > 0.05) of the CFI extract could be inferred.

## 4. Discussion

### 4.1. Lipid Oxidation Development in the Present Study

Lipid oxidation of seafood and food in general is considered a complex deteriorative mechanism involving the formation of a wide range of compounds. Being unstable, some of such compounds would be susceptible to breakdown and/or reaction with other molecules that are present in the fish muscle, especially with those presenting nucleophilic behaviour [45,46,47]. When a thermal treatment is concerned, as in the current study, different factors may influence the content of the different lipid oxidation compounds [6,7]. On one side, the heat treatment would give rise to an increased formation of lipid oxidation molecules; this event would be especially likely to occur based on the fact that the lipid composition of marine species includes a great content of highly unsaturated lipids [1,3,41]. Secondly, the thermal treatment itself would accelerate the breakdown of oxidation compounds originated in the first stages into lower-molecular-weight molecules (i.e., aldehydes, ketones, etc.), which are more reactive than primary molecules. Finally, following the formation and breakdown of lipid oxidation molecules (electrophilic-type molecules), the interaction of the resulting molecules with nucleophilic compounds (i.e., protein-type compounds) present in the fish muscle would accelerate the formation of fluorescent compounds.

According to the above-mentioned first effect, the present results have shown a relevant increase in the content of the primary lipid oxidation compounds measured (i.e., CDs, CTs, and peroxides) in the first stages of the heating time (0–5-day period). However, as the heating time increased, the second factor (thermal breakdown of oxidised compounds) was shown to increase its influence on the primary oxidation development. Thus, a marked peroxide content decrease was detected in the CTR batch for the 5–12-day period and in all CFI-treated batches for the 8–12-day period. This decrease was found more important in the CTR batch than in those including the CFI extracts. This difference can be justified by the presence in the ink extract of antioxidant compounds that would lead to an inhibitory effect on the breakdown of the primary oxidation compounds.

According to the above-mentioned third effect (i.e., interaction compound formation), the present results have shown a strong interaction between oxidised lipids and nucleophilic-type molecules present in the fish muscle. This interaction could be proved in the FR_org_ and the FR_aq_ values, both values increasing with the heating time. Notably, this FR increase was partially inhibited by the ink extract presence in the reacting medium; indeed, an increasing inhibitory effect was proven by increasing the ink extract concentration.

Most research regarding heat-treated seafood has shown low values for primary lipid oxidation compounds, indicating a marked effect of the thermal breakdown on primary compounds. Such research has concerned heated–minced mackerel (*Scomber scombrus*) (50 °C for 11 days) (low CD, CT, and peroxide contents) [13] and different kinds of canned seafood like bluefin tuna (*Thunnus thynnus*) and sardine (*Sardina pilchardus*) (low peroxide content) [42], Atlantic mackerel (*S. scombrus*) (low CD, CT, and peroxide contents) [30], Atlantic Chub mackerel (*Scomber colias*) (low peroxide content) [12], and eel (*Anguilla anguilla*) (low peroxide content) [48]. However, and according to the present results, a relevant increase in the primary compound contents has been detected in a heated marine oil system (increase in peroxide content) [11] and in cooked salmon (increase in peroxide content) (*Salmo salar*) pasta [49].

Regarding the determination of the fluorescent compound formation during the thermal processing of marine species, a relevant increase in the FR has been proved in a wide range of studies including fish model systems [11,13] and canned seafood [12,31,39,40]. Thus, the detection of such kinds of compounds was shown to be a valuable analytical procedure for quality loss assessment during seafood processing (chilling, frozen storage, and canning) [35,46].

A similar trend was obtained in the present study for the FR_org_ and the FR_aq_ determinations (Figure 1 and Figure 2). However, it has to be considered that both values provide different but complementary information regarding the interaction compound formation, i.e., lipophilic and hydrophilic molecules, respectively [11,13,43]. Thus, interaction compounds having a remarkable lipophilic framework would be likely to remain in the organic phase during the lipid extraction. Contrarily, fluorescent substrates resulting from the interaction between oxidised membrane lipids and amino compounds would remain attached to the amino constituent and be present in the aqueous phase originated during the extraction of fish lipids [50,51]. In the current results, the formation of both kinds of fluorescent compounds has been shown to increase progressively during the whole heating period. Both determinations have provided a valuable tool for assessing the increased lipid damage during the present heating process.

Interaction compound formation has shown an important effect on the nutritional and sensory values of seafood. As a result of an increased formation, relevant losses of essential amino acid (lysine, methionine, etc.) contents, a decrease in the digestibility value, and a decrease in the sensory value (development of off-odour, off-flavour, and browning, and texture modification) have been reported to be produced [45,46,47]. Therefore, and according to the present results on fluorescent compound formation, it can be concluded that the nutritional and sensory qualities of thermally processed seafood are enhanced by the addition of the current ink extract.

The present results have shown scarce effects of the thermal treatment on the FA parameters measured. In agreement with this conclusion, previous canning of brine-canned sprat (*Clupeonella cultriventris*) (STFA, MUFA, PUFA, and total ω3 values) [43], water-packed Atlantic mackerel (*S. scombrus*) (PI) [30], and brine-packed Chub mackerel (*S. colias*) (PI) [12] did not provide a definite trend in the FA profile. Contrarily, a relevant decrease in the PI was observed in a heated cod liver oil system (15, 25, or 50 °C for 12 days) [11], in cooked (90 ± 5 °C for 30 min) salmon (*S. salar*) pasta [49], and in heated–minced Atlantic mackerel (*S. scombrus*) (50 °C for 11 days) [13].

### 4.2. Antioxidant Activity of Ink Extracts

The current study has shown a strong inhibitory effect of the ink extract on the lipid oxidation development of seabream muscle. Antioxidant properties of cuttlefish ink constituents and of other cephalopod species have already been reported, both in in vitro [52,53] and food preservation [27,29] studies. This activity has been reported to reside in melanin and melanin-free ink fractions [23]. Thus, Chen et al. [26] indicated that melanin of squid ink can act as a superoxide dismutase and would be able to prevent free radical production by catalysing oxygen to hydrogen peroxide. For the melanin-free ink, the antioxidant behaviour would be related to the hydroxyl groups of the L-DOPA and L-dopamine components, which are able to donate hydrogen atoms to any radical present in the reacting medium. Vate and Benjakul [27] analysed the composition of the melanin-free ink obtained from splendid squid (*Loligo formosana*) and proved that the highest antioxidant activity was found in fractions with molecular weight lower than 3 kDa; remarkably, the radical scavenging activity remained constant if the ink extract was subjected to heat treatment (90 °C up to 30 min). Based on thin-layer chromatography analysis, Senan [24] indicated that the active principle in the cuttlefish (*Sepia pharaonis*) ink extract would be a peptide compound; this study also showed that the active principle was thermally stable (40–100 °C for 30 min). Similarly, previous research had already indicated that smaller-size peptides would be more stable in response to aggregation and high temperatures than bigger ones [54]. Recently, Tian et al. [55] proved the antioxidant behaviour of low-molecular-weight polysaccharides obtained from cuttlefish (*Sepiella maindroni*) ink. These three studies [24,54,55] demonstrated the interest in employing an aqueous extract of CFI, as in the current study, with the aim of increasing the rancidity stability of thermally treated seafood.

Taking into account all these characteristics of ink constituents, a wide range of previous in vitro studies have shown the antioxidant properties of cuttlefish and squid inks. The present results are in agreement with such inhibitory effects; however, CFI has not been employed previously with thermally treated seafood, to the best of our knowledge. Thus, based on the determination of the DPPH radical scavenging activity, a remarkable antioxidant behaviour was observed for cuttlefish (*S. officinalis)* [18,53], squid (*Loligo duvauceli*) [56], and cuttlefish (*Sepia esculenta*) [52]. Vate and Benjakul [27] showed that melanin-free ink obtained from splendid squid (*L. formosana*) had antioxidant activity according to different in vitro assays such as DPPH, ABTS, and ferric reducing power; additionally, this ink substrate showed a metal-chelating activity. Antioxidant properties (DPPH, FRAP assays) in melanin-free squid (*Loligo duvaucelli*) ink were also detected by Karim et al. [25].

Previous studies regarding the preservative effect of CFI extracts have also been focussed on non-thermally treated seafood. Thus, lipid oxidation (detection of peroxides and TBARS) was retarded in minced mackerel (*Rastrelliger kanagurta*) during 15-day storage in ice by the presence of melanin-free ink obtained from splendid squid (*L. formosana*) [27]. Similarly, Vate et al. [28] reported that melanin-free ink splendid squid (*Loligo formosana*) prevented lipid oxidation (detection of peroxides, TBARS, nonanal and 2-decenal) in surimi gel sardine (*Sardinella albella*) during storage at 4 °C for 20 days. An increased shelf-life time during cold storage (15 days at 4 °C) was detected in squid (*L. duvaucelli*) by previous squid melanin-free ink soaking [25]; additionally, an inhibitory effect on the trimethylamine (TMA) and total volatile base (TVB) values was detected. An inhibitory effect on microbial (mesophilic and psychrotrophic counts) activity and the formation of off-odour compounds (TMA and TVB values) due to previous cuttlefish (*S. officinalis*) ink soaking was proven in cold-stored (12 days at 0 °C and 23 days at −2 °C) peeled shrimp (*Penaeus kerathurus*) [57]. The shelf-life time of smoked sardine (*Sardinella aurita*) during cold storage (4 °C for 35 days) was increased by previous soaking in cuttlefish (*Sepia officinalis*) ink solution [53]; additionally, an inhibitory effect on lipid oxidation development (peroxide detection) and microbial activity (total viable count increase and formation of TVB and TMA) was proven.

### 4.3. Lipid Hydrolysis Development during the Present Study

In the current study, the FFA content measured can be influenced by different factors. First, the thermal process can lead to the hydrolysis of higher-molecular-weight lipid classes such as triacylglycerols (TAGs) and phospholipids (PLs) [6,9]. Secondly, FFAs are likely to be oxidised or broken down during the thermal treatment as they provide greater accessibility to oxygen and other pro-oxidant molecules when compared to the TAG and PL classes [58,59]; consequently, this effect would produce a decrease in the FFA value. Finally, the presence of antioxidant molecules included in the ink extract may protect FFAs from oxidation and breakdown processes; therefore, the content decrease resulting from the above-mentioned second effect could be decreased. Since samples corresponding to the CTR batch show increasing values with heating times, it could be concluded that the first factor was more relevant than the second one. However, the fact that comparison among batches did not lead to a definite trend indicates that the preserving effect of the ink extract was not strong enough to partially inhibit the FFA breakdown.

According to the present results, an increased FFA content has been described for thermally treated fish. This FFA formation concerns heated fish muscle systems such as cooked yellowfin tuna (*Thunnus albacares*) [60] and minced Atlantic mackerel (*S. scombrus*) (50 °C for 11 days) [13]. Regarding canned-fish systems, an increased FFA content as a result of the sterilisation process was detected in brine-canned silver carp (*H. molitrix*) [61]; sunflower- or brine-packed sprat (*C. cultriventris*) [43]; sunflower oil-, coconut oil-, and groundnut oil-packed yellowfin tuna (*T. albacares*) [60]; and sunflower oil-, soybean oil-, olive oil-, sunflower oil-, olive oil-, and spiced olive oil-packed eel (*A. anguilla*) [48].

No previous studies regarding the effect of ink extracts on the FFA content of thermally processed seafood are available, to the best of our knowledge. However, and contrary to the present results, previous research accounts for the FFA preservation in thermally treated seafood through the presence of antioxidant extracts obtained from other natural resources. Thus, the addition of macroalgae *Ulva lactuca* or *Fucus spiralis* extracts to the packing medium of canned Atlantic Chub mackerel (*S. colias*) led to FFA values of 13.4 and 12.8 g·kg^−1^ lipids, respectively, that were found remarkably higher than in samples corresponding to the control condition (9.1 g·kg^−1^ lipids) [12]. In a heated–minced mackerel (*S. scombrus*) system (50 °C for 11 days) [13], the presence of a red alga flour extract led to an FFA level of 5.6 g·kg^−1^ lipids at the end of the experiment; this value was notably higher than that in the case of the control (i.e., 1.0 g·kg^−1^ lipids).

## 5. Conclusions

The antioxidant properties of cuttlefish (*S. officinalis*) ink were analysed and demonstrated in a heated fish system. The presence of an ink aqueous extract led to a lower (*p* < 0.05) development of lipid oxidation (CD, CT, and fluorescent compounds) and to a higher (*p* < 0.05) retention of unsaturated FAs (PI determination). Preservative effects were found more relevant with an increase in the CFI concentration and the heating times. Contrarily, a definite effect on the FFA content (lipid hydrolysis development) could not be concluded.

This research provides a first approach for the beneficial employment of cuttlefish ink as a new preserving strategy for thermally treated seafood. It agrees with present general interests in food technology in the search for new tools including preservative compounds obtained from natural sources. Additionally, the current study contributes to achieving alternative sources for obtaining highly valuable compounds from seafood waste with the aim of providing increased profitability in using such biomass. According to the direct relationship between rancidity stability and nutritional and sensory values, it is considered that this study provides a new strategy for the quality enhancement of thermally treated seafood. Furthermore, the physical and sensory properties of the thermally treated seafood ought to be taken into account in order to ensure consumer acceptance of the resulting product.

Further research is found necessary to analyse and describe the active molecules involved. In order to optimise the extractability of the preservative molecules present in the current cuttlefish ink, a study on the comparative yield of hydrophilic (water) and lipophilic (organic solvents) extracts is found necessary. With this purpose, the employment of advanced extraction procedures (microwave-, ultrasound-, supercritical-, or high-pressure-assisted) ought to be addressed too.

## Figures and Tables

**Table 1:** Primary lipid oxidation development * in seabream muscle heated in the presence of an aqueous extract of cuttlefish ink (CFI) **.

Quality Index	Heating Time (Days)	CFI Concentration			
		CTR	CFI-1	CFI-2	CFI-3
Conjugated diene formation ***	0	0.27 a (0.01)	0.27 a (0.01)	0.27 a (0.01)	0.27 a (0.01)
	2	0.39 bB (0.05)	0.31 bA (0.01)	0.30 bA (0.00)	0.31 bA (0.00)
	5	0.50 cB (0.04)	0.35 cA (0.02)	0.33 cA (0.01)	0.33 bA (0.01)
	8	0.53 cB (0.02)	0.52 dB (0.04)	0.41 dAB (0.08)	0.38 cA (0.04)
	12	0.46 bcA (0.05)	0.49 dA (0.04)	0.46 dA (0.02)	0.44 cA (0.05)
Conjugated triene formation ***	0	0.02 a (0.00)	0.02 a (0.00)	0.02 a (0.00)	0.02 a (0.00)
	2	0.04 aA (0.01)	0.03 aA (0.00)	0.03 aA (0.00)	0.03 aA (0.00)
	5	0.07 bB (0.01)	0.04 abA (0.01)	0.04 aA (0.00)	0.04 aA (0.00)
	8	0.11 cB (0.01)	0.08 bcAB (0.03)	0.05 abA (0.03)	0.04 abA (0.01)
	12	0.10 bcA (0.02)	0.09 cA (0.02)	0.08 bA (0.01)	0.08 bA (0.02)
Peroxide value (meq active oxygen·kg^−1^ lipids)	0	0.50 a (0.10)	0.50 a (0.10)	0.50 a (0.10)	0.50 a (0.10)
	2	10.72 bC (1.74)	4.14 bB (1.01)	2.13 bAB (0.94)	1.59 bA (0.38)
	5	32.19 dC (3.82)	7.63 cB (1.45)	5.11 cB (1.90)	2.71 cA (0.22)
	8	24.61 cA (3.18)	40.11 eB (4.78)	25.51 eA (2.14)	24.55 eA (2.12)
	12	9.07 bA (4.49)	22.11 dB (2.74)	21.24 dB (2.06)	16.77 dAB (4.75)

* Mean values of three replicates (*n* = 3); standard deviations are shown in brackets. Different lowercase letters (a–e) denote differences (*p* < 0.05) as a result of the heating time. Different capital letters (A–C) indicate differences (*p* < 0.05) as a result of the CFI concentration. ** Abbreviations: CTR (Control batch); CFI-1, CFI-2, and CFI-3 correspond to batches including low, medium, and high CFI concentrations, respectively. *** Units as expressed in the Section 2.

**Table 2:** Fatty acid (FA) ratios * in the lipid fraction of seabream muscle heated in the presence of an aqueous extract of cuttlefish ink (CFI) **.

FA Ratio	Heating Time (Days)	CFI Concentration			
		CTR	CFI-1	CFI-2	CFI-3
Polyene index	0	0.56 b (0.01)	0.56 a (0.01)	0.56 a (0.01)	0.56 a (0.01)
	2	0.56 bA (0.01)	0.56 aA (0.01)	0.56 aA (0.00)	0.56 aA (0.01)
	5	0.54 abA (0.01)	0.56 aAB (0.00)	0.57 aB (0.01)	0.56 aAB (0.00)
	8	0.53 aA (0.01)	0.55 aAB (0.01)	0.56 aAB (0.02)	0.56 aB (0.01)
	12	0.53 aA (0.01)	0.54 aA (0.01)	0.56 aA (0.02)	0.55 aA (0.02)
Total MUFAs + Total PUFAs/Total STFAs	0	3.48 (0.11)	3.48 (0.11)	3.48 (0.11)	3.48 (0.11)
	2	3.49 (0.17)	3.47 (0.27)	3.49 (0.05)	3.49 (0.09)
	5	3.45 (0.26)	3.47 (0.11)	3.49 (0.15)	3.49 (0.08)
	8	3.42 (0.15)	3.45 (0.47)	3.47 (0.44)	3.46 (0.16)
	12	3.44 (0.26)	3.43 (0.31)	3.45 (0.11)	3.46 (0.39)
Total ω3 FAs/Total ω6 FAs	0	0.40 (0.01)	0.40 (0.01)	0.40 (0.01)	0.40 (0.01)
	2	0.40 (0.01)	0.40 (0.01)	0.39 (0.01)	0.40 (0.02)
	5	0.39 (0.01)	0.40 (0.01)	0.41 (0.01)	0.40 (0.01)
	8	0.39 (0.01)	0.40 (0.01)	0.40 (0.02)	0.40 (0.01)
	12	0.40 (0.01)	0.40 (0.01)	0.41 (0.01)	0.40 (0.02)

* Mean values of three replicates (*n* = 3); standard deviations are shown in brackets. Different lowercase letters (a,b) denote differences (*p* < 0.05) as a result of the heating time. Different capital letters (A,B) denote differences (*p* < 0.05) as a result of the CFI concentration. No differences (*p* > 0.05) in MUFA + PUFA/STFA and ω3/ω6 ratios were detected. ** Abbreviations: monounsaturated FAs (MUFAs), polyunsaturated FAs (PUFAs), saturated FAs (STFAs); CFI concentrations as expressed in Table 1.

## Data Availability

All data are contained within the article.
